# Supplementary material for: Patients with central serous chorioretinopathy have high circulating alpha-klotho concentrations
Source: PLoS One. 2023 Dec 21;18(12):e0295795. doi: 10.1371/journal.pone.0295795 (PMC10734912; doi:10.1371/journal.pone.0295795)
Supplement: Attachment — Submitted filename: Response to Reviewers yuki yamamoto.docx [file pone.0295795.s002.docx]

Dear Dr. Chenette,

Thank you for taking the time to review this manuscript. We appreciate the prompt review and the opportunity to revise and improve the manuscript further. We are grateful to the two reviewers for the time and effort that they have spent reviewing the manuscript. Please find our point-by-point responses to the comments below. The text shown in red below is a copy of the modified parts of the manuscript, and these are shown as tracked changes in the revised version, which is attached.

**Responses to** **Reviewer #1**

**#1. There is insufficient information provided regarding the study design. For example, a more detailed description of inclusion and exclusion criteria, the number of patients excluded, and how subjects were selected is needed. How was randomization performed? There is also no mention of the timing of blood sampling or whether treatment was performed for acute or chronic CSC, among other things**

Response: We apologize for not fully describing the study design. We thank the reviewer for raising this issue. The study was retrospective in nature, as described on line 67 and randomization was not performed.

With respect to the inclusion and exclusion criteria, we have added the following explanation (Lines 74–80, page 5):

“We included patients with both acute and chronic CSC, which we categorized based on a duration of symptoms of less or more than 6 months, respectively. Patients with macular neovascularization detected using optical coherence tomography angiography (OCTA) at baseline; those who had used steroids; those with systemic diseases, such as renal or cardiac disorders, which affect αKl concentration; those with a history of anti-vascular endothelial growth factor drug therapy; and those with severe myopia of >−6.0 D were excluded.”

Five patients with macular neovascularization, two with steroid use, two with systemic disease, and three with strong myopia were excluded.

We have added the following regarding the timing of the blood sampling:

“To ensure uniformity of the conditions, after a diagnosis of CSC had been made by FA and ICGA, blood samples were drawn approximately 6 hours after waking from patients who gave their consent, and at the time consent was given in controls” (Lines 87–92, page 5-6).

We have also added the initial treatments administered to lines 101–104 page 6.

**#2. In this study, there is a significant difference in smoking history as a background factor. Smoking is a known risk factor for CSC. Therefore, the author should be matched as a background factor.**

Response: We apologize for not matching the patients with regard to their smoking history. To account for potential confounding by the association between smoking and CSC, variables potentially associated with smoking, including sex and age, were included in a logistic regression model in which smoking was the dependent variable, to calculate a propensity score for CSC. A propensity score-matched subpopulation was then selected from the study sample by matching patients with CSC and controls who were smokers. The αKI concentrations were then compared between these two groups, and the αKI concentration was found to be significantly higher in the patients with CSC (*p*=0.033).

We have added the following explanation to lines 134–138, page 7:

“A propensity score-matched subpopulation was selected from the study sample by matching patients with CSC and controls according to smoking status. The αKI concentrations of patients with CSC and normal controls were compared, and the αKI concentration was found to be significantly higher in patients with CSC (*p*=0.033).”

**#3. The recurrence of CSC cases was also evaluated in this study, and it is necessary to describe whether there is a history of treatment for CSC and the treatment methods used.**

Response: We thank the reviewer for their recommendation. Of the 10 cases of recurrence, one eye had a previous history of CSC, and the patient showed spontaneous remission. However, we did not have records of any previous history of CSC, but instead received self-reports at the patient’s first visit to our clinic. Therefore, this information may not have been very accurate.

We have added the following explanation to lines 168–169, page 9:

Of the 10 cases of recurrence, one eye had a previous history of CSC, and the patient showed spontaneous remission.

**#4. The author should evaluate the relation between smoking and αKl in recurrence cases.**

Response: We thank the reviewer for this suggestion. In cases of recurrence, three of the 10 eyes were in patients with a smoking habit. In cases of recurrence, there were no significant differences in αKI by smoking habit status.

We have added an explanation to the manuscript as follows (lines 174–175, page 10):

“In cases of recurrence, there were no significant differences in αKI by smoking habit status..”

**#5. The authors may also need a larger sample size. If the sample size increased, the statistical results will be different and became not significant.**

Response: We agree that the relatively small sample size was the major limitation of the study. We had aimed to collect 50 samples from both CSC and controls; however, after matching the participants and applying the exclusion criteria, the number was lower in the normal group. In response to this comment, we have strengthened our discussion of the limitations of the study.

We have added the following explanation (Lines 247–250, page 13):

“We had aimed to collect 50 samples from both CSC and controls; however, after matching and the application of the exclusion criteria, the number of controls available for inclusion was lower. If the sample size were to be increased, the significance levels associated with the results would likely change.”

**#6. The author described that no significant relationship was found between αKI concentration and SFCT in the analysis of all cases of CSC, but what about in the case of recurrent CSC?**

Response: We thank the reviewer for this constructive remark. There was no significant relationship between αKl and SFCT in participants who experienced recurrence (*p*=0.999).

We have added the following explanation (Lines 175–176, page 10):

“Similarly, no significant difference in αKI concentrations and SFCT in patients with recurrent cases. (*p*=0.999)

”

We thank the reviewer for the comments.

**Responses to** **Reviewer #2**

1. **It would be interesting to compare CSC patients with no history of smoking (n=20) and control subjects with no history of smoking (n=23), because alpha-klotho is reported to reflect smoking status.**

Response: We thank the reviewer for carefully reading the manuscript and the suggestion. We have performed an analysis of patients who were and were not smokers in the CSC and control groups, and the αKl concentration was found to be significantly higher in the CSC group (888.9 ± 80 and 727.6 ± 92pg/mL, respectively; *p* = 0.017).

We have added this finding to lines 163–166, page 9, as follows:

“However, a comparison of the αKl concentrations between patients with CSC and normal controls who were not smokers showed that they were significantly higher in the CSC group (888.9 ± 80.0 and 727.6 ± 92.0 pg/mL, *p* = 0.017).

**2. Is there any correlation between alpha-klotho and other biomarkers in the systemic circulation? There are several systemic diseases reported to affect alpha-klotho, most of which also influence, or are influenced by, systemic biochemical markers.**

Response: We thank the reviewer for this question.

We apologize that we only checked the status of both patients with CSC and normal controls with respect to the presence of underlying disease, such as heart failure and kidney dysfunction, and the use of oral medication in the present study. In addition, we recorded each patient’s medical history but did not evaluate heart failure or renal function using laboratory investigations. Previous studies have shown that αKI is associated with both renal and heart failure. As the reviewer points out, we could have made interesting findings if we had measured parameters related to renal function and blood pressure, for example.

We comment regarding hypertension below.

**3. There are some reports stating that hypertension is related to CSC. Please show blood pressure or a history of hypertension in this case series.**

Response: We thank the reviewer for this constructive remark.

Blood pressure was measured in all of the patients at the time of the FA/ICGA, and there were 17 patients with a systolic blood pressure of ≥140 mmHg, who were defined as having hypertension. When these patients were compared with the patients with CSC who did not have hypertension, no significant difference in αKl concentration was identified (*p*=0.668).

We have added this finding to lines 189–194, page 10, as follows:

“Blood pressure measurements were taken before FA/ICGA for CSC patients, we examined whether there was a difference in αKI according to blood pressure. There were 17 patients with a systolic blood pressure of ≥140 mmHg, who were defined as having hypertension. When these patients were compared with the patients with CSC who did not have hypertension, no significant difference in αKl concentration was identified (*p*=0.668)

We thank the reviewer for the comments.

We have attempted to improve the manuscript in accordance with the reviewers’ comments. We sincerely appreciate their constructive comments and hope that the revised manuscript will now be acceptable for publication in *PLOS ONE.* We would like to thank you for permitting us to submit a revised version of the manuscript.

Yours sincerely,

Yuki Yamamoto
